# Development Temperature Has Persistent Effects on Muscle Growth Responses in Gilthead Sea Bream

**DOI:** 10.1371/journal.pone.0051884

**Published:** 2012-12-17

**Authors:** Daniel Garcia de la serrana, Vera L. A. Vieira, Karl B. Andree, Maria Darias, Alicia Estévez, Enric Gisbert, Ian A Johnston

**Affiliations:** 1 Physiological and Evolutionary Genomics Laboratory, Scottish Oceans Institute, School of Biology, University of St Andrews, St Andrews, Scotland, United Kingdom; 2 Institut de Recerca i Tecnologia Agroalimentàries, Sant Carles de la Ràpita, Catalonia, Spain; Centre of Marine Sciences & University of Algarve, Portugal

## Abstract

Initially we characterised growth responses to altered nutritional input at the transcriptional and tissue levels in the fast skeletal muscle of juvenile gilthead sea bream. Fish reared at 21–22°C (range) were fed a commercial diet at 3% body mass d^−1^ (non-satiation feeding, NSF) for 4 weeks, fasted for 4d (F) and then fed to satiation (SF) for 21d. 13 out of 34 genes investigated showed consistent patterns of regulation between nutritional states. Fasting was associated with a 20-fold increase in *MAFbx*, and a 5-fold increase in *Six1* and *WASp* expression, which returned to NSF levels within 16h of SF. Refeeding to satiation was associated with a rapid (<24 h) 12 to 17-fold increase in *UNC45*, *Hsp70* and *Hsp90α* transcripts coding for molecular chaperones associated with unfolded protein response pathways. The growth factors *FGF6* and *IGF1* increased 6.0 and 4.5-fold within 16 h and 24 h of refeeding respectively. The average growth in diameter of fast muscle fibres was checked with fasting and significant fibre hypertrophy was only observed after 13d and 21d SF. To investigate developmental plasticity in growth responses we used the same experimental protocol with fish reared at either 17.5–18.5°C (range) (LT) or 21–22°C (range) (HT) to metamorphosis and then transferred to 21–22°C. There were persistent effects of development temperature on muscle growth patterns with 20% more fibres of lower average diameter in LT than HT group of similar body size. Altering the nutritional input to the muscle to stimulate growth revealed cryptic changes in the expression of *UNC45* and *Hsp90α* with higher transcript abundance in the LT than HT groups, whereas there were no differences in the expression of *MAFbx* and *Six1*. It was concluded that myogenesis and gene expression patterns during growth are not fixed, but can be modified by temperature during the early stages of the life cycle.

## Introduction

The gilthead sea bream (*Sparus aurata*) is widely cultivated in the Mediterranean with an annual production of 125,000 metric tonnes in 2010 [1]. High rates of growth are a priority in intensive aquaculture systems. The main non-genetic factors influencing growth rate are body mass, diet composition, ration size, stocking density and other environmental factors such as temperature, water quality and oxygen availability [2]. Juvenile gilthead sea bream (1–10 g) can achieve growth rates of 5% d^−1^ at the optimal temperature of 21–22°C doubling in body mass every 1–2 weeks. The growth of fast skeletal muscle in juvenile fish involves the proliferation of a resident population of Pax7 expressing myogenic progenitor cells (MPCs) to produce myoblasts committed to terminal differentiation (reviewed in [3], [4]). The MyoD family of muscle transcription factors play a pivotal role in the commitment (MyoD, Myf5, MRF4) and differentiation (MRF4, Myogenin) of myoblasts [5]. Some MPCs fuse to form myotubes which initiate myofibrillargenesis and sarcomere assembly and mature into new muscle fibres (reviewed in [3], [4]). Others are absorbed into existing muscle fibres to maintain the nuclear to cytoplasmic ratio within certain limits as fibres increase in length and diameter with growth [6]. Many transcription factors and signaling molecules regulating muscle growth have been described; however the detailed genetic and cellular mechanisms linking body mass, temperature and feeding intensity remain to be elucidated. Insulin-like growth factor (IGF1 and IGF2) signalling is involved in regulating many aspects of myogenesis including myoblast proliferation and the balance between protein synthesis and translation which determines hypertrophic growth [4]. The liver is the main site for the production of circulating IGF1 under the influence of growth hormone produced by the hypophysis [7]. In addition, *IGF1* is expressed in most tissues including skeletal muscle indicating the presence of both paracrine and endocrine regulatory networks [8]. The IGF system comprises several membrane receptors, and six IGF binding proteins (IGFBPs) which regulate the effective concentration of the hormone with some components existing as paralogues due to genome duplication in the teleost lineage [9]. Feeding promotes protein accretion through phosphorylation of key components of the IGF-signaling pathway including the target of rapamycin (mTOR) and Akt which activate translation [8]. Phosphorylation of Akt also inhibits the ubiquitin proteasome protein degradation pathway by phosphorylation of Forkhead box-O (FOXO) transcription factors causing them to become translocated out of the nucleus inhibiting the ubiquitin ligases Murf1 and MAFbx [10]. Studies of fasting and refeeding have shown that the muscle transcriptome is also exquisitely sensitive to nutritional input, reflecting the anabolic or catabolic status of the fish [11]. Significant changes in transcript levels can occur very rapidly e.g. Atlantic salmon (*Salmo salar*) fed a single satiating meal after a period of fasting produced a peak in muscle *IGFBP4* expression within 1 h of feeding [12]. This is particularly relevant during periods of compensatory growth, which are described as the exceptionally fast growth that occurs after a period of moderate or severe reduction in feeding. This phenomenon is normally associated with increased food ingestion rates and sometimes improved food conversion efficiency (reviewed in [13]).

Embryonic temperature change (ET) produces persistent changes in growth and muscle cellularity in teleosts grown at a common temperature from hatching [14], [15]. For example, in the zebrafish *Danio rerio*, the lifetime production of fast muscle fibres showed an optimum at 26°C ET and was 19% and 14% lower at 22°C ET and 31°C ET respectively [16]. Embryonic temperature was also shown to alter swimming performance with temperature acclimation in adult zebrafish, producing changes in muscle fibre composition and the expression of 61 out of 32,988 unique transcripts quantified by RNAseq [17].

The objective of the present study was to explore thermal imprinting of muscle growth in the gilthead sea bream. First we developed a compensatory growth model which explored the time course of responses to altered food intake. Early events investigated included phosphorylation of AKT, a key control protein in IGF-signaling, and the transcriptional regulation of genes associated with protein folding (*Hsp90α, Hsp90β, Hsp30, Hsp70, UNC45, eIF2a*), growth regulation (*MAFbx*, *CathepsinD1, Calpain-3, IGF1, AKT2, IGFBP4, PCNA, Follistatin, FGF6, GHR1, Erk2, IGF2*) or myogenesis (*MyoD1, MyoD2, Myf5, MRF4, Myogenin, Caveolin-3, Six1, MEF2C, NAFTC2, m-cadherin, STAC3, Sox8, Pax7, WASp, CAMKII*). Later events investigated included the abundance of myogenic progenitor cells and the production, and hypertrophy, of fast muscle fibres. We established that early temperature treatments altered muscle cellularity in juvenile fish reared at a common temperature. Using the fasting-feeding model we then tested the hypothesis that early temperature experience had persistent effects on gene expression patterns, providing a potential mechanism contributing to altered growth responses in the muscle.

## Results

### Characterisation of Growth Responses to Altered Nutritional Input

The average growth rate with non-satiation feeding (NSF) was 2.52% body mass d^−1^ (N = 60). In fish fasted for 4d the gastrointestinal tract was completely empty and the gall bladder was massively extended with bile. This period of fasting reduced the hepatosomatic index (HSI) by 70% from 2.4±0.18% to 0.68±0.07%, to increase again during SF (Mean ± SE, N = 15, P<0.01) (Figure S1). Refeeding to satiation resulted in a transitory (<48 h) 2-fold increase in gut food content relative to NSF (10.0±0.4% versus 5.0±0.6%, Mean ± SE, N = 15, P<0.01) and HSI returned to NSF levels by 24 h (Figure S1). The average growth rate with SF was 5.25% body mass d^−1^ or 2-times the NSF rate with fish increasing in mass by 2.8-fold after 12d SF (N = 60) (Figure S1).

**Figure 1:**
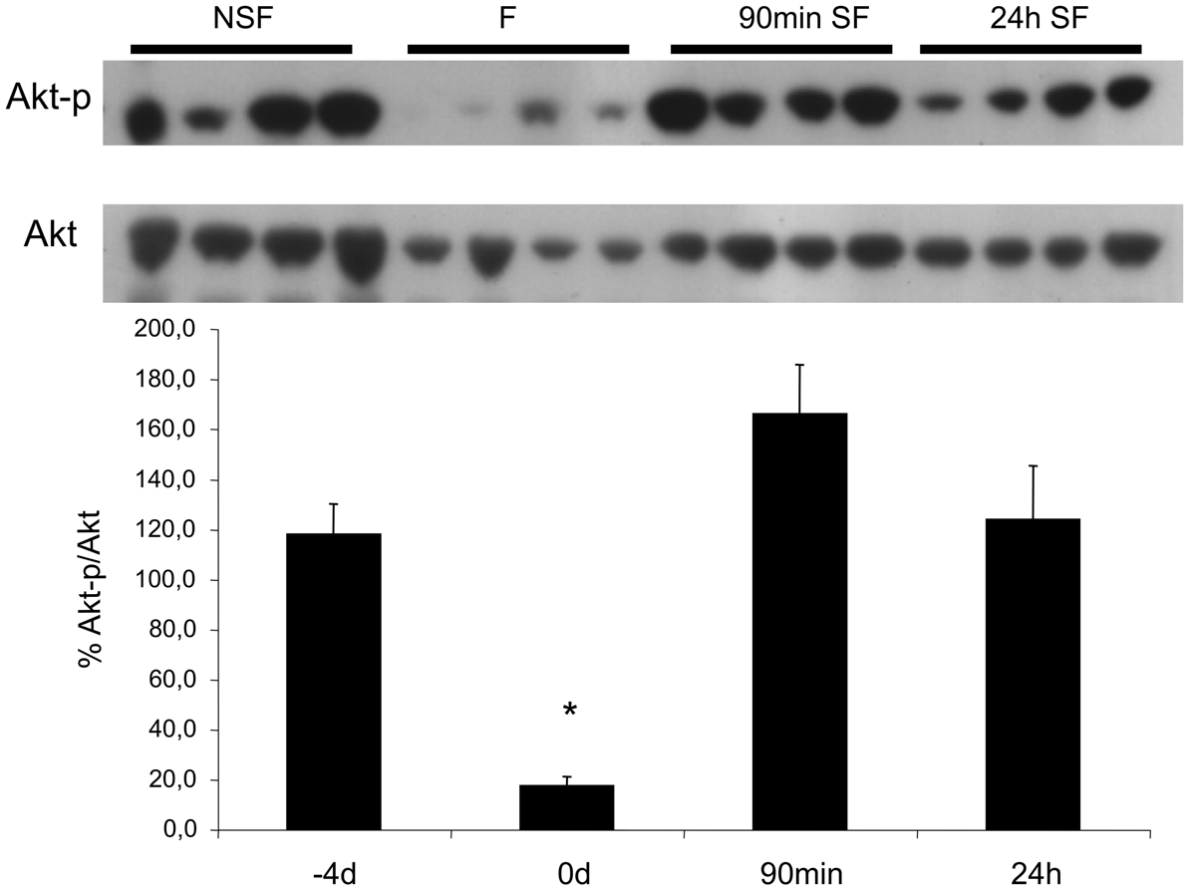
Phosphorylation of the insulin-like growth factor (IGF) pathway protein AKT in skeletal muscle. (Top) A representative western blot for phospho-Akt during non-satiation feeding (NSF, −4d), after 4 days of fasting (F, 0d), 90 minutes and 24 hours of satiation feeding (SF). (Bottom) Percentage phosphorylation of Akt, see text for details of normalisation method. Asterisk denotes significant differences between treatments (P<0.05).

The percentage phosphorylation of Akt, a regulator of Insulin-like Growth Factor signalling, was decreased by 60–70% between the NSF and fasted states, but was not significantly elevated in the early stages of refeeding to satiation (Figure 1).

**Figure 2:**
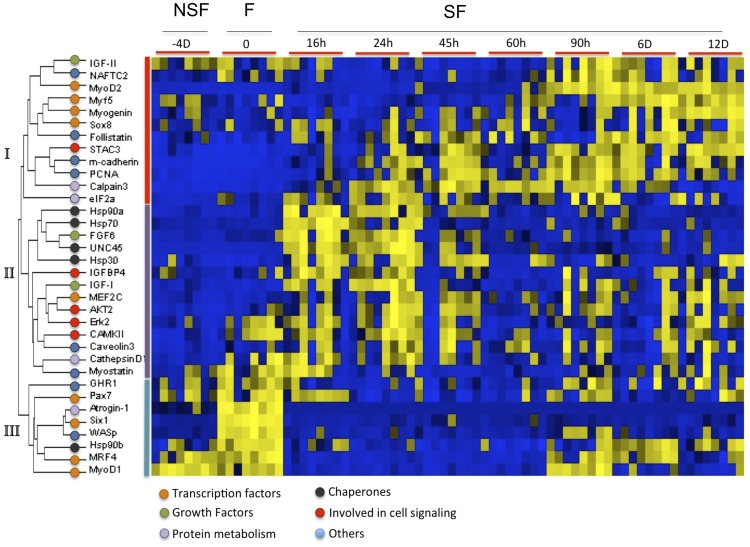
Heat map summary and hierarchical cluster for the 34 genes analysed in fast muscle. Genes shown were analysed during transition from non-satiation fed (NSF), fasting (F) and satiation fed (SF). Rows are standardized to have a mean of 0 and standard deviation of 1; yellow indicates high and blue indicates low expression values. SF = Satiation feeding, F =  fasting, NSF = Non-satiation feeding. Insulin-like growth factor 1 (*IGF1*), Myostatin (*MSTN*), myoblast determination factor 2 (*MyoD2*), growth hormone receptor 1 (*GHR1*), myogenic regulator factor 4 (*MRF4*), insulin-like growth factor 2 (*IGF2*), paired box transcription factor 7 (*Pax7*), myogenic factor 5 (*Myf5*), sex determination region Y box 8 (*Sox8*), myogenic regulator factor 1 (*MyoD1*), heat shock protein 70 (*Hsp70*), heat shock protein 30 (*Hsp30*), heat shock protein 90 alpha (*Hsp90*α), heat shock protein 90 beta (*Hsp90*β), SH3 and cysteine rich domain 3 (*STAC3*), proliferating cell nuclear antigen (*PCNA*), insulin-like growth factor binding protein 4 (*IGFBP4*), mitogen activated protein kinase (*Erk2*), v-akt murine thymoma viral oncogene homolog 2 (*AKT2*), muscle cadherin/cadherin 15 (*m-cadherin*), myocyte enhancer factor 2c (*MEF2C*), nuclear factor of activated T-cells calcineurin depenent 2 (*NFATC2*), Atrogin-1 (*MAFbx*), eukaryotic initiation translation factor 2a (*eIF2a*), Wisskott-Aldrich syndrome protein (*WASp*), sine oculis homeobox 1 (*Six1*), fibroblast growth factor 6 (*FGF6*) and calcium/calmodulin-dependent protein kinase 2 (*CAMKII*). Colour circles indicate different functional categories, orange = transcription factors, green = growth factors, pink = protein metabolism, black = chaperones, red = cell signaling and blue = others. Roman numbers indicate the three main groups of genes formed during the analysis.

Nested ANOVA with tank as random effect showed that transcripts for 30 of the 34 genes showed no tank effect and they were combined for statistical analysis. *MRF4*, *PCNA*, *m-cadherin* and *CAMKII* showed a relatively small, but statistically significant tank effect (P = 0.05–0.04). A summary heat map and unsupervised hierarchical clustering of gene expression following modification of the nutritional input to the muscle is shown in Figure 2. An unsupervised hierarchical cluster for “time-point” was also performed and revealed discrete clusters for fasted and fed fish (Figure S2). 13 genes showed consistent patterns of regulation between nutritional states (*IGF1*, *FGF6*, *MyoD1*, *MyoD2*, *Hsp90α*, *UNC45*, *Hsp70*, *Calpain*-*3, Myogenin*, *Myf5*, *MAFbx*, *WASp* and *Six1*) whereas 21 genes exhibited stochastic expression both between individual fish and between time points (*NAFTC2*, *Hsp90β*, *GHR1*, *IGF2*, *MRF4*, *Pax7*, *Myostatin*, *CathepsinD1*, *Caveolin-3*, *PCNA*, *m-cadherin*, *STAC3*, *Hsp30*, *eIF2a*, *AKT2*, *Erk2*, *Sox8*, *Follistatin*, *IGFBP4*, *CAMKII, MEF2C*). The 13 nutritionally responsive genes were also measured at four time points in replicate tanks of fish continuously fed 3% body mass d ^–1^. For 11 of the 12 genes there was no significant difference in expression levels over 12d (e.g. Hsp90α in Figure S3A). MyoD2 showed a significant increase in expression with continuous feeding after 12d, but the effect was much reduced relative to the fasting-feeding experiment (Figure S3B).

**Figure 3:**
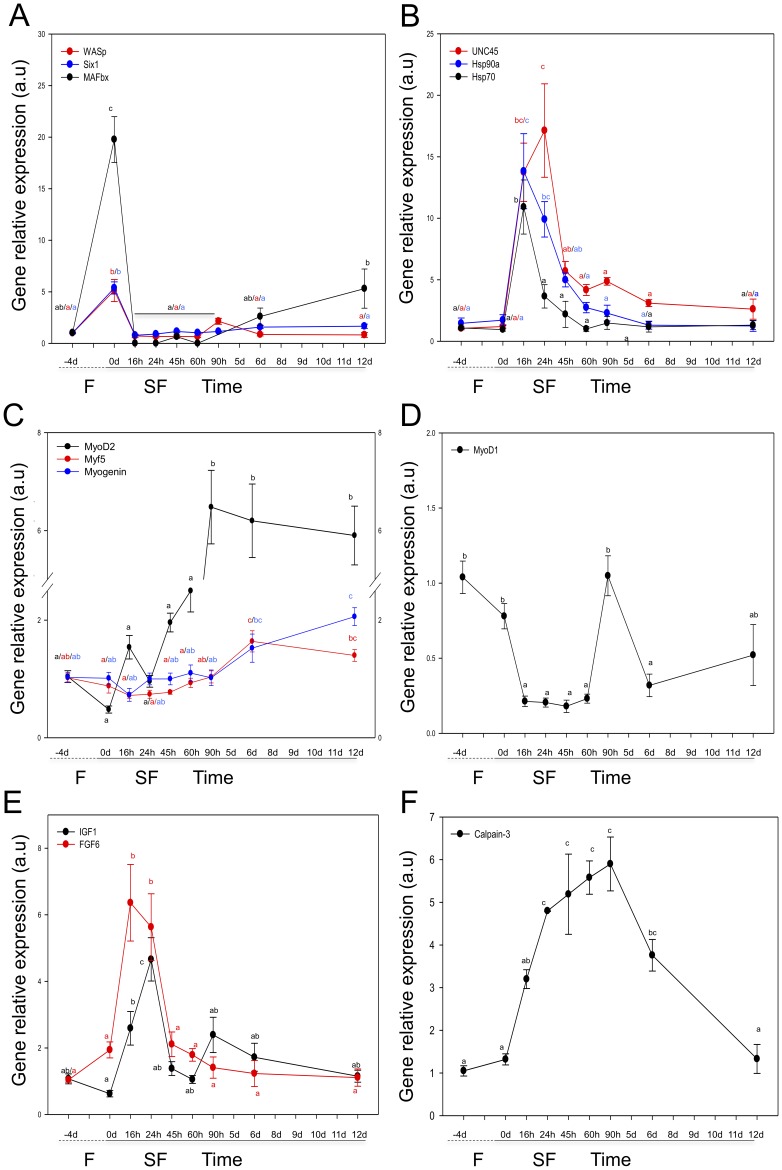
Relative expression of genes consistently affected by nutritional state. Effect of varying nutritional input on gene expression in skeletal muscle during 4 days of fasting (F, dashed line) and 12 days of satiation feeding (SF, solid line). Values represent mean ± SE (N = 8). Different black, red and blue letters indicate significant differences between mean values (P<0.05). A) *MAFbx* (black), *Six1* (blue) and *WASp* (red), genes up-regulated with fasting B) Molecular chaperones, *Hsp70* (black), *UNC45* (red) and *Hsp90α* (blue), C) Myogenic Regulatory Factors, *MyoD2* (black), *Myf5* (red) and *Myogenin* (blue), D) *MyoD1*, E) Growth Factors, *IGF1* (black) and *FGF6* (red), F) Ca^2+^-activated protease, *Calpain-3*.

Fasting resulted in a 20-fold increase in *MAFbx*, and a 5-fold increase in *Six1* and *WASp* expression, which returned to NSF levels within 16 h (Figure 3A). Refeeding to satiation was associated with a rapid (<24 h) 12 to 17-fold increase in *UNC45*, *Hsp70* and *Hsp90*α transcripts which code for molecular chaperones associated with the unfolded protein response (Figure 3B). The expression of myogenic regulatory factors varied with altered nutritional input. *MyoD2* expression was similar between the NSF and fasting states, but increased significantly from 90 h until 12d after refeeding (P<0.01), as did *Myf5* and *Myogenin* expression (Figure 3C). In contrast *MyoD1* showed a complex expression profile with lower levels of expression during the early stage of refeeding (Figure 3D). The expression of the growth factors *FGF6* and *IGF1* increased 6.0-fold and 4.5-fold within 16 h and 24 h of refeeding (P<0.01) respectively and then declined to NSF levels after 48 h (Figure 3E). *Calpain-3* expression increased 5–6 fold at 24 h SF and was maintained until 6d (P<0.01) (Figure 3F). Expression profiles and statistical analysis of the 34 genes investigated can be found in File S1.

**Figure 4:**
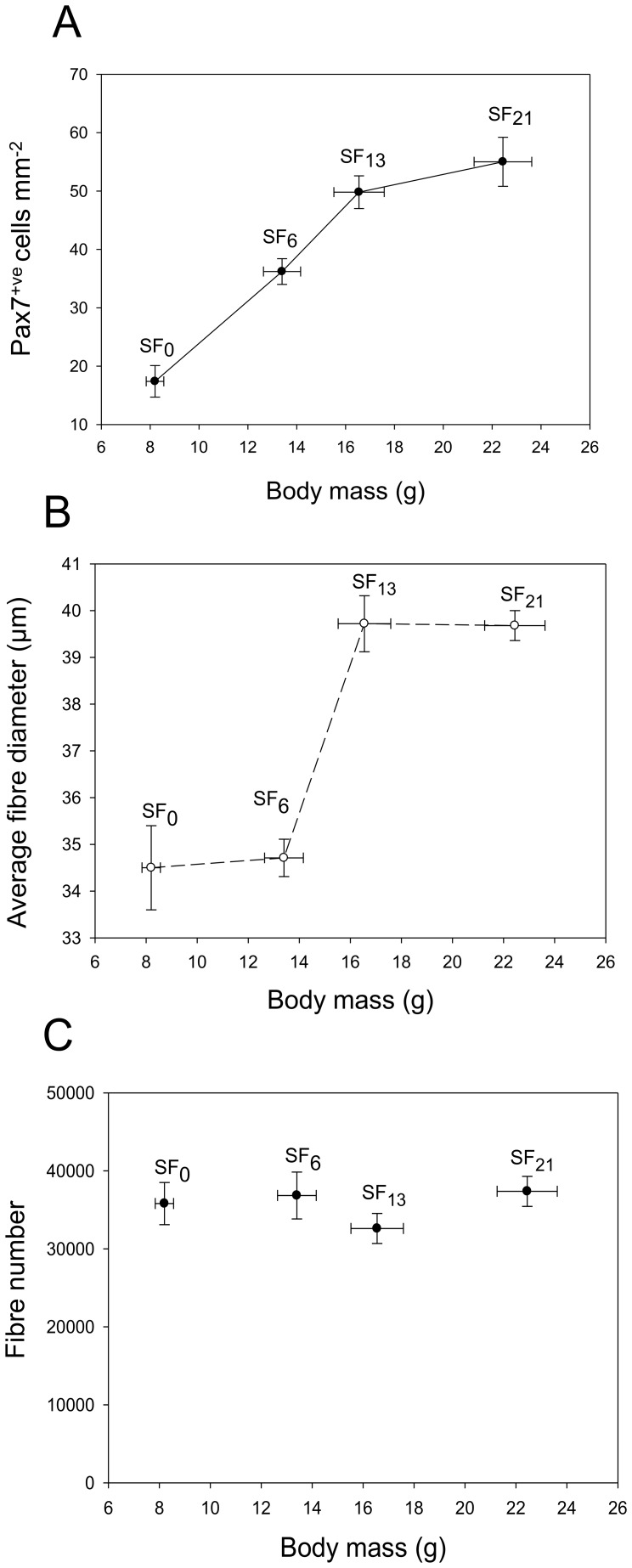
Muscle cellularity following fasting (SF_0_) and satiation feeding (SF_6–12_). A) Immunohistochemistry for Pax7^ve+^, a marker for myogenic progenitor cells, B) average fibre diameter (µm) and C) muscle fibre number per myotomal cross-section. Values represent mean ± SE, N = 12 fish per sample.

*Pax7* expression was 2-fold higher in the fasting than NSF and SF samples (P<0.05) (File S1). The density of myogenic progenitor cells identified by staining with a Pax7 antibody increased over 2-fold during refeeding (Figure 4A) and preceded fibre hypertrophy. The average diameter of fast muscle fibres was checked with fasting and a significant increase in hypertrophic growth with refeeding was only observed after 13d and 21d (Figure 4B). Although body mass increased 2.8-fold and muscle cross-sectional area by 38% (not shown) with 21d SF this was not associated with a statistical increase in the number of fast muscle fibres (Figure 4C) which averaged 37,400±2,400 (Mean ± SE, N = 8, all SF samples combined).

### Early Temperature Treatments Affected Juvenile Muscle Growth

Eggs incubated at 21–22°C (HT) had a medium time to hatching of 42 h compared to 48 h at 17.5–18.5°C (LT). By 69 days post-hatching (dph) both groups had metamorphosed, at an average length of around 17 and 21 mm for the LT and HT treatments respectively. Fish were transferred to a common temperature of 21–22°C at 101 dph at the early juvenile stage. At transfer average body mass was significantly higher in the HT (2.9±1.1 g) than in the LT group (1.2±0.4 g) (Mean ± SD, N = 60, P<0.001). After being transferred to the same temperature the LT group showed compensatory or “catch-up” growth such that at 202 dph there was no significant difference between temperature treatments (Figure S4) with no significant tank effect.

In juveniles matched for similar overall body size at 202 dph, the LT group had 20% more fast myotomal muscle fibres per cross-section (90,900±2,400) than the HT group (75,700±6,000 (P = 0.015) (Mean ± SE, N = 6 per treatment). In contrast, the average fibre diameter was significantly less in the LT (42.8±1.3 µm) than HT treatments (49.2±1.6 µm) (P<0.05) (Mean ± SE, N = 6 per treatment).

### Early Temperature Treatments Modified Gene Expression

The expression of selected genes in the HT and LT groups was investigated using the feeding-fasting protocol characterised above. Gut content and HSI showed a similar pattern with respect to nutritional regime for all temperature treatments (Figure S5).

**Figure 5:**
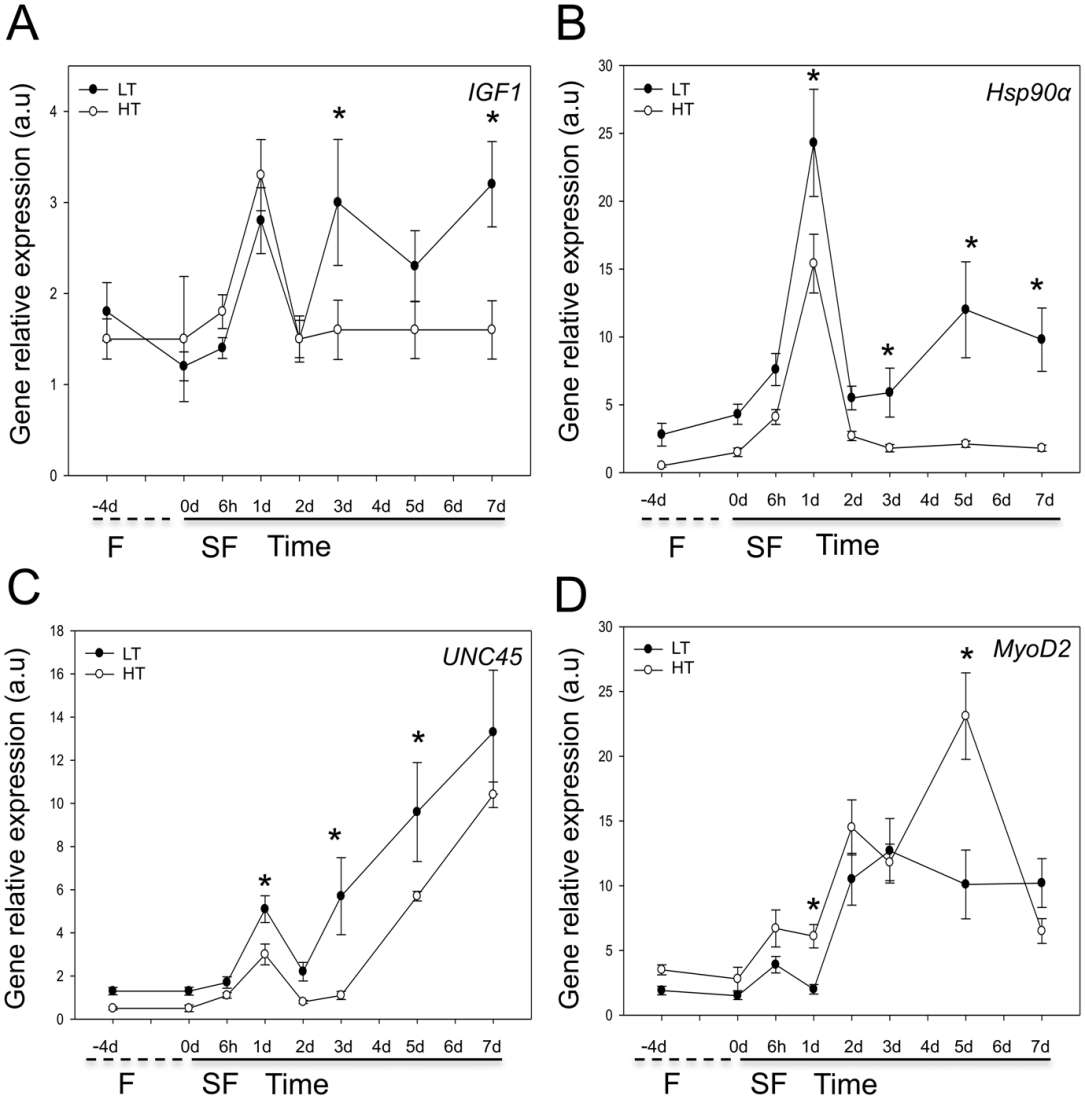
Gene expression of gilthead sea bream juveniles reared at 17.5–18.5°C (low temperature treatment, LT) and 21–22°C (high temperature treatment, HT) prior to the early juvenile stage. Fish were fasted for 4d (F, dashed line) and satiation fed for 7 (SF, solid line). Values represent mean ± SE, (N = 12 fish per time point for each treatment). Asterisk shows significant differences in mean values between LT (filled circles) and HT (open circles) treatments at each time point A) *IGF1* B) *Hsp90α* C) *UNC45* D) *MyoD2*.

No tank effect was found in gene expression data. The expression of *MAFbx*, *Six1* (Figure S6) and *MyoD2* (Figure 5D) was significantly correlated with gut fullness (statistics are summarized at Table S1) and showed a similar pattern to that obtained previously with fish reared at a constant temperature of 21–22°C from fertilisation (P = 0.00) (Table S1). Four of the six genes investigated, *IGF1* (figure 5A), *Hsp90α* (Figure 5B), *UNC45* (Figure 5C) and *MyoD2* (Figure 5D) showed significant differences in expression between the HT and LT groups reflecting persistent influences of early temperature regime (Table S1). *Hsp90*α and *UNC45* showed higher expression at all nutritional states in the LT than HT groups (Figure 5B, C). After 1d and 5d satiation feeding transcript levels in the LT than HT groups were 24-fold and 15.4-fold higher respectively for *Hsp90α* and 9.6 and 5.7-fold higher respectively for *UNC45* (Figure 5B and C) (P<0.05). In contrast, *IGF1* transcript levels were similar between temperature groups with NSF and fasting, but 3–3.2 fold higher in the LT than HT groups with 3–7d satiation feeding (P<0.05) (Figure 5A).

## Discussion

Manipulation of feeding status and diet composition has been commonly used to study growth responses and anabolic to catabolic transitions in fish [13], [18], [19]. In the present study, we exploited the recently published gilthead sea bream fast muscle transcriptome [20] to investigate the expression of 34 candidate growth related genes in fish that were fed 3% body mass d^−1^, fasted for 4d and then fed to satiation to initiate a compensatory growth response. We choose a relatively short fasting period which was sufficient to elevate expression of *MAFbx* indicating activation of the ubiquitin-proteasome system and to elicit a strong transcriptional response to satiation feeding, but which did not result in a change in muscle water content, indicating it was probably insufficient to result in muscle atrophy. In contrast, many previous gene expression studies of this kind have used longer fasting periods of several weeks which resulted in transcriptional responses associated with muscle atrophy and a prolonged and relatively slow return to growth with satiation feeding, presumably because of the need for a period of recovery and repair [11].

The large decrease in HSI we observed in gilthead sea bream indicates that energy reserves are preferentially mobilised from the liver during the initial phases of fasting in this species. *MAFbx* a sensitive indicator of the transition from an anabolic to catabolic state increased more than 20-fold with a 4d fast (Figure 3A). Fasting was also associated with a marked increase in *Six1* expression a homeobox transcription factor previously shown to impair myogenesis in zebrafish embryos after knockdown with anti-sense morpholinos [21]. Studies in adult mice suggested that Six1 drives the switch from a slow-fibre to a fast-fibre phenotype [22]. Recent findings using C2C12 mouse cell line suggested that an over-expression of *Six1* reduced myoblast proliferation [23]. Therefore, Six1 may have a role in regulating myoblast proliferation and/or fibre phenotype in the fasted state.

We found large and transitory increases in the expression of chaperones involved in protein folding, *Hsp90α*, *Hsp70* and *UNC45*, within 16 h of satiation feeding, consistent with a rapid initiation of protein synthesis (Figure 3B). Similar activation of protein unfolding response pathways has been reported in salmonids during catabolic-anabolic transition [11]. UNC45 is known to specifically interact with Hsp90α to control the correct folding of myosin heavy chains [24]. The initial increase in protein synthesis is thought to result from the increased phosphorylation of a number of proteins in the IGF-signalling pathway, including Akt [8]. Significant increases in Akt phosphorylation had occurred by the first sample point within 90 min of feeding (Figure 1). Circulating IGF1, produced by the liver binds to IGF receptors in the muscle sarcolemma [25] and is also synthesised locally by autocrine pathways [8]. *IGF1* transcripts increased 4-fold by 16 h and 7-fold by 24 h after resumption of satiation feeding, consistent with a strong and relatively long lasting activation of autocrine IGF production (Figure 3E). Phosphorylation of FOXO proteins by IGF1 downregulates *Murf1* and *MAFbx* [26] and transcripts for *MAFbx* had decreased 20-30-fold to almost undetectable levels by 16 h (Figure 3A). Studies in mammals suggested that *FGF6* expression regulates muscle differentiation and the maintenance of muscle integrity during mechanical stress [27]. *FGF6* showed rapid (<24 h) and transitory increases with SF (Figure 3E). Previous studies in rainbow trout (*Oncorhynchus mykiss*) reported no change in *FGF6* expression with fasting followed by satiation feeding [28], although fish were first sampled after 4d and therefore may have been missed the early responses of this growth factor to SF. Calpain-3 is involved with sarcomere remodelling during muscle fibre growth [29] and transcripts for this gene were upregulated from 45 h after feeding (Figure 3F). Although the processes of myofibrillargenesis and sarcomere assembly appear to be initiated within a few days significant increases in fibre diameter were only observed after 13d (Figure 4). Fibre hypertrophy is associated with accretion of nuclei derived from Pax7^+ve^ MPCs [15]. *Pax7* mRNA was higher in fasted than fed individuals whereas MPCs expressing Pax7 protein increased with satiation feeding, with a significant difference occurring after 6d, prior to significant fibre hypertrophy (Figure 4B). Although we assume MPCs also fused to form new myotubes over the time scale of the experiment this was not sufficient to produce a significant increase in fibre number (Figure 4C).

The gilthead sea bream genome has two *MyoD* paralogues (*MyoD1 and MyoD2*) arising from whole genome duplication at the base of the teleost radiation [30]. MyoD2 is fast skeletal muscle-specific whereas MyoD1 is present both in fast and slow skeletal muscle [31]. MyoD is an important member of the myogenic regulatory factors (MRFs) family of transcription factors with pivotal roles in myogenic determination and differentiation [4]. Atlantic salmon has lost *MyoD2*, but has three *MyoD1* paralogues (*MyoD1a, MyoD1b, MyoD1c*) due to salmonid-specific whole genome and local duplication events [30]. In synchronised primary myogenic cultures *MyoD1a* and *MyoD1c* were modulated in phase with the cell cycle whereas *MyoD1a* expression was strongly correlated with *Myogenin* expression and terminal differentiation [32]. Depriving Atlantic salmon cells of amino acids led to an increase in *Pax7* and an increase in *MyoD1c* and *PCNA* expression indicating a transition to a quiescent state [8]. In the gilthead sea bream, experimentally induced fasting *in vivo* also led to an increase in *Pax7* transcripts and high *MyoD1* expression, which was strongly downregulated in the early stages of SF treatment, a similar response to that described *in vitro* in Atlantic salmon [12]. In contrast, *MyoD2* expression peaked after 3d SF and remained elevated along with *Myogenin* and *Calpain-3* (Figure 3C and F), consistent with its role in terminal differentiation fulfilling a similar function to *MyoD1a* in Atlantic salmon.

In gilthead sea bream, the proportion of growth in muscle girth arising from hyperplasia and fibre hypertrophy varied with the environmental temperature in the early stages of the life cycle. Fish from the LT group had more muscle fibres per myotomal cross-section of smaller average diameter than fish from the HT group. Similar thermal imprinting phenomena have been reported in a wide range of fish species [15]. The lifetime production of fast muscle is also influenced by embryonic temperature exhibiting a bell-shaped reaction norm with an optimum near the mid-point of the temperature range for normal embryonic temperature e.g. 5°C in Atlantic salmon [33] and 26°C in zebrafish [16]. There is some evidence in the European sea bass (*Dicentrarchus labrax*) that temperature variation in later life history stages e.g. warming during seasonal cooling in juveniles can also affect the production and size of muscle fibres [14]. In the case, of gilthead sea bream the highest production of fast fibres in juveniles was observed for a pre-metamorphosis temperature of 18°C which is towards the lower limit for normal reproduction and development [34]. In Atlantic salmon [33] and gilthead sea bream (present study) fish reared at the optimal temperature for fibre production had a smaller body size at transfer to common temperature than fish reared at warmer temperatures, but showed compensatory or catch-up growth during subsequent stages. We tested the hypothesis that thermal imprinting of growth capacity altered transcriptional responses in our fasting-feeding model. Four out of the six genes studied had significantly different expression profiles, consistent with widespread and persistent effects of early temperature regime on gene expression. *HSP90α* and *UNC45* showed higher expression in all nutritional states, consistent with higher rates of protein synthesis and protein folding activity in the LT than HT treatments. Since the food content of the gut following satiation feeding was similar between temperature groups this suggests that higher heat shock protein expression in LT than HT fish was not due to greater food consumption or changes in amino acid absorption capacity at the level of the gastrointestinal system. *IGF1* transcript levels were higher following 3d SF in the LT than HT groups, but not at other nutritional states, consistent with a higher local production of *IGF1* contributing to the compensatory growth response (Figure 5A). Differences in *MyoD2* expression between temperature groups were complex and less easy to interpret. Although only a small number of genes were studied this was sufficient to confirm our hypothesis of persistent effects of early temperature regime on juvenile gene expression. Similar results were recently obtained from a genome-wide survey of gene expression in zebrafish using RNAseq [17]. In this study, embryonic temperatures (ET) at the limits for normal development were found to improve swimming performance following cold acclimation relative to fish raised at optimum ET, and this was associated with altered expression of more than 50 genes associated with energy metabolism. Thus in two unrelated species early thermal environment has been shown to have persistent effects on gene expression and physiological performance in adult fish. The mechanism(s) are unknown but could include epigenetic modification of chromatin e.g. by DNA methylation or selection through differential survivorship of offspring, particularly in gilthead sea bream which under captive rearing conditions can show 75–85% larval mortality. From the perspective of aquaculture and conservation restocking programs these results clearly demonstrate that species-specific responses to environmental conditions in the hatchery have significant long-term consequences for the growth performance of adult stages.

### Conclusions

Transcript levels of many growth-related genes in fast muscle, including *MAFbx*, *Six1*, *MyoD2*, heat shock protein genes and *Calpain-3* were strongly correlated with gut fullness, responding to altered nutritional input within 16 h. Early temperature regime had persistent effects on muscle cellularity in juvenile fish and modified the expression of a sub-set of genes (*Hsp90α, UNC45, MyoD2, IGF1*) during compensatory growth experiments at a common temperature. We conclude that muscle growth does not follow a fixed pattern, but rather can be modified by the temperature experienced during early stages of the life cycle.

## Materials and Methods

### Fish Husbandry

Juvenile gilthead sea bream (*Sparus aurata* L.) around 6 g body mass were maintained in duplicate tanks at the Institute for Aquaculture and Food Technology Research (IRTA) St Carles de la Ràpita, Spain, in a temperature-controlled seawater re-circulation system (IRTAmar™) at 21–22°C with a natural photoperiod for September (13L: 11D). Fish, 1000 per tank (initial stocking density = 4.1 kg m^−3^), were fed 3% (m/m) d^−1^ of a commercial diet (OptiBream™, Skretting, Norway) for 4 weeks (Non-satiation feeding, NSF) and then fasted for 4d (F) and re-fed to satiation for 21d (Satiation feeding, SF) using a combination of automatic feeders and hand feeding. Simultaneously, a group of gilthead sea bream were continuously fed at 3% (m/m) d^−1^ of a commercial diet (OptiBream™) and maintained in duplicated tanks in similar conditions of fish density, temperature and photoperiod. Fish were sacrificed according to the veterinary committee of IRTA in accordance with EU regulations (EC Directive 86/609/EEC) and body mass and fork length (FL, tip of snout to fork in the tail) recorded to the nearest 0.01 g and 1 mm, respectively. Fast skeletal muscle from dorsal epaxial myotomes at 0.5 FL, liver and the gastrointestinal system were dissected on a pre-chilled glass plate at 0–4°C. Muscle samples were flash frozen in liquid nitrogen and stored at −80°C until further analysis. Samples were taken after 4 weeks NSF (−4d), 4d fasting (0d) and 16 h, 24 h, 45 h, 72 h, 4d, 6d, and 13d SF, plus 21d for histological analysis. Animals continuously fed at 3% (m/m) d^−1^ were sacrificed at equivalent time-points: 0d, 45 h, 6d and 13d.

To generate temperature treatments, 440 ml of fertilized eggs (fertilization rate = 92%) were maintained during embryogenesis at 17.5–18.5°C (LT) or 2122°C (HT) in duplicate conditions (110 ml of fertilized eggs per incubator). Each incubator (35 l) consisted of a mesh basket (150 µm pore size) equipped with an airlift that was submerged in a 2,000 l tank connected to a re-circulation system in order to maintain stability of water quality parameters within the optimal ranges for the species [34]. Embryogenesis was continuously monitored under the microscope. Hatching rates were 71.0±5% and 74.0±8% for LT and HT groups, respectively. Once larvae hatched, they were distributed in two 2,000 l tanks and reared until the juvenile stage under standard conditions. Following the completion of metamorphosis (101 dph) fish from both treatments were transferred to a common temperature regime of 21–22°C for 202 days prior to initiation of the experiment. During this period animals were maintained at the same biomass density and fed 3% (m/m) d^−1^ of a commercial diet (OptiBream™). Fish of similar size from both groups were then sampled after fasting for 4 days and satiation feeding (SF) for 12 h, 1d, 2d, 3d, 5d and 7d. The hepatosomatic index (HSI, %) was calculated as the liver weight as a percentage of the whole body mass.

Changes in growth rates over time were calculated by means of the specific growth rate (% day^−1^) defined as SGR = 100 * (ln BW_f_ – ln BW_i_) days^−1^, where BW_f_ and BW_i_ were the final and initial fish body weights between sampling points (N = 30 fish per tank, 60 fish per treatment). The mesenteric fat index (MFI, %) was calculated as the gut fat as a percentage of the whole body mass. Relative gut content was defined as GT = 100 * (full gut weight – empty gut weight)/BW_f_. Condition factor (CF) was calculated as CF = BW_f_*100/L^3^ where L was the length of the animal. Spleen body index (%) was calculated as the spleen weight as a percentage of the whole body mass. The average body mass of fish in temperature treatments was determined from measurements of 30 fish per tank (60 fish per treatment).

### RNA Extraction and cDNA Synthesis

RNA was extracted using QIAzol (QIAGEN, Crawley - West Sussex, UK) following the manufacturer’s recommendations. The integrity of the RNA was confirmed by ethidium bromide gel electrophoresis. RNA concentration, 260/280 and 260/230 ratios were evaluated using a NanoDrop 1000 spectrophotometer (Thermo Fischer Scientific, Waltman, MA). All RNA samples had a 260/280 nm ratio higher than 1.9 and 260/230 ratio above 2.2. Genomic DNA was removed using genomic DNA wipe-out buffer included in the Quantitect reverse transcription kit (Qiagen). RNA (1 µg) was reverse transcribed into cDNA for 30 min at 42°C using a Quantitect reverse transcription kit following the manufacture’s recommendations. To ensure that no genomic DNA was present in the samples a control without the reverse transcriptase enzyme was performed.

### Quantitative Real-time PCR

The following procedures were compliant with the minimal information requirements for publication of quantitative PCR guidelines [35].

Primers were designed using Net primer (Premier BioSoft) to have a TM of 60°C, and where possible to cross an exon-exon junction. For primer design of genes *AKT2*, *CAMKII*, *m-cadherin*, *PCNA*, *Six1*, *WASp*, *MAFbx*, *Erk2*, *Calpain-3*, *Caveolin-3*, *STAC3*, *CathepsinD1*, *eI2F*, *NAFTC2*, *MEF2C*, *Hsp90*β, *Hsp90*α, *IGFBP4* and *FGF6* sequences were retrieved from the available gilthead sea bream skeletal muscle transcriptome (SRA accession number: ERA047531; File S2) [20] (sequences retrieved are summarized in File S2). The primers, amplicon size, amplicon melting temperature and accession numbers of genes for qPCR are listed in Table S2. Quantitative PCR (qPCR) were performed using a qPCR machine (MX3005P, Stratagene, La Jolla, CA, USA) and Brilliant II SyberGreen (Stratagene). Each qPCR reaction contained 7.5 µl of Brilliant II SYBR Green Master Mix, 6 µl cDNA (80-fold dilution) and 0.75 µl of each primer at 500 nM to a final volume of 15 µl. Amplification was performed in duplicate in 96-well plates (Stratagene) with the following thermal cycling conditions: initial activation 95°C for 10 min followed by 40 cycles of 15 s at 95°C, 30 s at 60°C, and 30 s at 72°C. Control reactions included a no-template control and retrotranscription control (RT). To analyse dissociation of the PCR products, a gradient from 60°C to 95°C was run to confirm the presence of a single PCR product. Products were confirmed by direct sequencing using the University of Dundee Sequencing service. A dilution series (1∶20, 1∶40, 1∶80, 1∶160, 1∶320, and 1∶640) of pooled cDNA samples was used to calculate PCR amplification efficiencies (Table S2).

Four reference genes r18, β-actin, elongation factor 1 alpha (eF1α) and ribosomal protein L27 (RPL27) (Table S2) were tested for stability using GeNorm software [36]. GeNorm analysis showed that RPL27 (M score = 0.29) was the most stable reference gene and this was used for normalisation using the Pfalff method [37]. Gene expression was normalised to the NSF sample.

### Protein Extraction

Fast muscle (40 mg) was homogenized in a Lysis Matrix-D tube (MP Biomedicals, Irvine, CA, USA) using 500 µl of RIPA buffer (SIGMA, Dorset, UK) containing a mix of phosphatases and proteases inhibitors (SIGMA). The homogenate was incubated for 1 h at 4°C with continuous agitation and the supernatant was collected after centrifugation of 10 min at 13000 rpm. Protein concentration was measured using the Bradford method [38].

### Histological Analysis

For histological analysis a 5 mm thick transversal section was cut at ∼0.7 FL as measured from the snout, and a high-resolution photograph taken to determine the cross-sectional area of fast muscle. Histological samples were taken 4d before fasting (−4d), 4d after fasting (0d), and 6d, 13d and 21d refeeding. For the temperature experiments, fish were sampled prior fasting (−4d) and at the beginning of refeeding period (0d). 2–3 blocks, sufficient to sample one half of the myotomal cross-section were mounted on cork strips and frozen in isopentane cooled to its freezing point in liquid nitrogen. 7 µm thick frozen sections were cut on a cryostat (Leica Microsystems, CM1850, Milton Keynes, UK). Sections were mounted in poly-L-lysine coated slides and stained with Harris’ modified hematoxylin (SIGMA) for fibre visualization. The total cross sectional area of the trunk was determined from digital photographs of sections. Approximately 1000 fibres evenly distributed among blocks, were quantified for each fish using AxiaVision 4.3.1 software (Carl Zeiss MicroImaging, Cambridge, UK). Muscle fibre number and diameter and Pax7^+ve^ myogenic progenitor cells were determined as previously described [39].

### Western Blot Analysis

A quantity of 20 µg of protein was added to 3 µl of 5X protein loading buffer and 1 µl of 20X reducing agent (Fermentas, Vilnius, Lithuania) and RIPA buffer to 15 µl. Samples were heated for 10 min at 95°C and loaded on to a NuPAGE® Novex 4–12% (m/v) poly-acrylamide gel (Invitrogen, Carlsbad, CA, USA). A pre-stained protein ladder ranging from 250 to 10 kDa (BioRad, Hemel, Hetfordshire, UK) was included in all gels to determine the molecular mass of bands. Samples were resolved by electrophoresis for 2 h at 100V and room temperature (RT). Proteins were then transferred to a PDVF Immobilon-P Transfer Membrane (Millipore, Billerica, MA, USA) at 25V for 2 h at RT. Membranes were washed twice with PBT (0.1% (v/v) Tween 20, SIGMA, in PBS) and blocked for non-specific binding with 5% (m/v) non-fat milk (AppliChem, Darmstadt, Germany) solution in PBT for 1 h at RT. After washing in PBT three times for 10 min membranes were incubated at 4°C overnight with the following primary antibodies: phospho-Akt (Ser473) (Cell Signaling, 4060, Danvers, MA, USA), Akt (Cell Signaling, 2966) and actin (SIGMA, A2066). Akt antibodies were diluted 1∶1000 (v/v) and actin in 1∶20000 (v/v) in PBT-0.01% (m/v) NaN_3_. Membranes were subsequently incubated with secondary anti-rabbit antibody linked to a horseradish peroxidase (HRP) (SIGMA) diluted 1∶40000 (v/v) in 5% (m/v) non-fat milk PBT solution for 1 h at RT. After washing in PBT three times for 15 min, membranes were incubated for 1 min with ECL Western Blot detection reagents (GE HealthCare, Amersham, Buckinghamshire, UK). Membranes were exposed to Hyperfilm ECL (GE HealthCare). The resulting films were scanned and band density evaluated with TotalLab Quant software (TotalLab, Newcastle, UK). Phospho-Akt levels were normalized with respect to the total Akt. A common pooled sample was loaded onto all gels for normalization.

### Statistical Analysis

Statistical analysis was performed using PASW Statistics 18.0 for Mac. When data on transcription and protein phosphorylation conformed to parametric assumptions a 1-way ANOVA, with tank as random effect was performed, followed by post-hoc tests with Bonferroni correction. When parametric assumption was not met, the Kruskal-Wallis test was used. Significant differences between treatments were accepted at the P<0.05 level. Unsupervised hierarchical clustering analysis of gene expression data was performed using Permutmatrix [40]. A multivariate linear model was used to study temperature imprinting on gene expression. *Hsp90*α, *UNC45*, *Six1*, *MAFbx*, *MyoD2* and *IGF1* were analysed using temperature as a fix factor, gut content and time were considered as co-variables with Bonferroni correction for the confidence interval. Also, 2-way ANOVA followed by a Bonferroni post-hoc correction was used with time and temperature as a factor to study differences between temperature treatments. The possibility of a tank effect in each treatment was tested again using 1-way ANOVA with time as the fixed factor and tank as the random factor. Significant differences between treatments were accepted at P<0.05. For the analysis of the fibre number and size a 1-way ANOVA, followed by post-hoc test with Bonferroni correction, was performed using MINITAB TM statistical software 13.20 (Minitab Inc.).

### Ethical Approval

Animal experimental procedures were conducted in compliance with the experimental research protocol (reference number 4978-T9900002) approved by the Committee of Ethic and Animal Experimentation of the IRTA and the Departament de Medi Ambient i Habitatge (DMAH, Generalitat de Catalunya, Spain) in accordance with EU regulation (EC Directive 86/609/EEC).

## Supporting Information

Figure S1 **Gilthead sea bream body parameters during fasting and refeeding.** Body size parameters in gilthead sea bream fasted for 4d (F, dashed line) and re-fed for 16 h, 24 h, 45 h, 60 h, 90 h, 6d and 12d (SF, solid line). Values represents mean ± SE (N = 60 for body mass, N = 15 for other parameters). Different letters indicate significant differences between means (P<0.05). A) Body mass (g) B) Hepatosomatic index (%) C) Relative gut content (%) after 4d of fasting (F) and 16 h, 24 h and 45 h of refeeding (SF) (filled circles) and during non-satiation feeding (NSF) (open circles).
(TIFF)

Figure S2 **Heat map summary and hierarchical cluster for the 34 genes analysed in fast muscle.** Unsupervised hierarchical cluster of 34 genes during fasting and refeeding. Data was clustered by “expression” and “time-point”. Rows are standardized to have a mean of 0 and standard deviation of 1; yellow indicates high and blue indicates low expression values. Insulin-like growth factor 1 (*IGF1*), Myostatin (*MSTN*), myoblast determination factor 2 (*MyoD2*), growth hormone receptor 1 (*GHR1*), myogenic regulator factor 4 (*MRF4*), insulin-like growth factor 2 (*IGF2*), paired box transcription factor 7 (*Pax7*), myogenic factor 5 (*Myf5*), sex determination region Y box 8 (*Sox8*), myogenic regulator factor 1 (*MyoD1*), heat shock protein 70 (*Hsp70*), heat shock protein 30 (*Hsp30*), heat shock protein 90 alpha (*Hsp90α*), heat shock protein 90 beta (*Hsp90β*), SH3 and cysteine rich domain 3 (*STAC3*), proliferating cell nuclear antigen (*PCNA*), insulin-like growth factor binding protein 4 (*IGFBP4*), mitogen activated protein kinase (*Erk2*), v-akt murine thymoma viral oncogene homolog 2 (*AKT2*), muscle cadherin/cadherin 15 (*m-cadherin*), myocyte enhancer factor 2c (*MEF2C*), nuclear factor of activated T-cells calcineurin depenent 2 (*NFATC2*), F-box protein 32 (*MAFbx*), eukaryotic initiation translation factor 2a (*eIF2a*), Wisskott-Aldrich syndrome protein (*WASp*), sine oculis homeobox 1 (*Six1*), fibroblast growth factor 6 (*FGF6*) and calcium/calmodulin-dependent protein kinase 2 (*CAMKII*).
(TIFF)

Figure S3 **Comparison of gene expression in gilthead sea bream either continuously fed at 3% body mass d^−1^ (NSF) or subject to 4 days fasting followed by satiation feeding (F-SF)** A) *Hsp90α* and B) *MyoD2*. CF (filled circles) and F-SF (open circles).
(TIFF)

Figure S4 **Comparison of body mass in gilthead sea bream reared at either 17.5–18.5°C (LT) or 21–22°C (HT) until 101d post-hatching and then transferred to a common temperature of 21–22°C.** LT (filled circles) or HT (open circles). The open arrow illustrates the end of metamorphosis in treatment groups (13–34 mm). The solid arrow indicates when treatment groups were transferred to a common temperature. Values represents mean ± SD (N = 60).
(TIFF)

Figure S5 **Comparison of body morphological parameters in gilthead sea bream reared at either 17.5–18.5°C (LT) or 21–22°C (HT) until 101d post-hatching and then transferred to a common temperature of 21–22°C.** Fish reared at either 17.5–18.5°C (filled circles) and 21–22°C (open circles) were fasted for 4d (F, dashed line) and re-fed to satiation (SF, solid line). A) Fork length (cm) B) Condition factor (%) C) Hepatosomatic index (%) D) Mesenteric fat index (%) E) Gut content (%) F) Spleen body index (%). Values represents mean ± SE (N = 12 fish per time point for each treatment).
(TIFF)

Figure S6 **Comparison of MAFbx and Six1 expression profiles in gilthead sea bream reared at either 17.5–18.5°C (LT) or 21–22°C (HT) until 101d post-hatching and then transferred to a common temperature of 21–22°C.** Gilthead sea bream juveniles reared at either 17.5–18.5°C (filled circles) and 21–22°C (open circles) were fasted for 4d (F, dashed line) and re-fed to satiation (SF, solid line). Values represent mean ± SE, (N = 12 fish per time point for each treatment). A) *MAFbx* B) *Six1*.
(TIFF)

Table S1 **Multivariate statistical analysis of body morphological and gene expression parameters in gilthead sea bream reared at either 17.5–18.5°C (LT) or 21–22°C (HT) until 101d post-hatching and then transferred to a common temperature of 21–22°C.** Dependent variables were hepatosomatic index (HSI), mesenteric fat index (MFI), *UNC45*, heat shock protein 90-alpha (*Hsp90α*), *MAFbx*, sine oculis homeobox homolog (*Six1*), Insulin-like growth factor 1 (*IGF1*) and myoblast determination protein (*MyoD2*) expression. Temperature was used as a fixed factor, gut content and time were considered as co-variables with Bonferroni correction for the confidence intervals.
(DOCX)

Table S2 **Primer design and qPCR parameters.** Forward and reverse primer sequences (5′–3′), amplicon product sizes in base pairs (bp), melting temperature of the amplicon (Tm), PCR efficiency (E), regression analysis of plasmid dilution series (R^2^) and identification of genes used in qPCR. Genes are as follow: Ribosomal protein L27 (*RPL27*), mitochondrial ribosomal protein S18 (*S18*), elongation factor 1-alpha (*EF1a*), insulin-like growth factor 1 (*IGF1*), Myostatin (*MSTN*), myoblast determination factor 2 (*MyoD2*), growth hormone receptor 1 (*GHR1*), myogenic regulator factor 4 (*MRF4*), insulin-like growth factor 2 (*IGF2*), paired box transcription factor 7 (*Pax7*), myogenic factor 5 (*Myf5*), sex determination region Y box 8 (*Sox8*), myogenic regulator factor 1 (*MyoD1*), heat shock protein 70 (*Hsp70*), heat shock protein 30 (*Hsp30*), heat shock protein 90 alpha (*Hsp90α*), heat shock protein 90 beta (*Hsp90β*), SH3 and cysteine rich domain 3 (*STAC3*), proliferating cell nuclear antigen (*PCNA*), insulin-like growth factor binding protein 4 (*IGFBP4*), mitogen activated protein kinase (*Erk2*), v-akt murine thymoma viral oncogene homolog 2 (*AKT2*), muscle cadherin/cadherin 15 (*m-cadherin*), myocyte enhancer factor 2c (*MEF2C*), nuclear factor of activated T-cells calcineurin depenent 2 (*NFATC2*), F-box protein 32 (*MAFbx*), eukaryotic initiation translation factor 2a (*eIF2a*), Wisskott-Aldrich syndrome protein (*WASp*), sine oculis homeobox 1 (*Six1*), fibroblast growth factor 6 (*FGF6*) and calcium/calmodulin-dependent protein kinase 2 (*CAMKII*), muscle specific calcium activated neutral protease 3 (*Calpain-3*), protein unc-45 (*UNC45*).
(DOCX)

File S1 **Expression profiles of 34 growth-related genes in gilthead sea bream skeletal muscle during fasting (F) and satiation feeding (SF).** Values represent mean ± SE (N = 8). Different letters indicate significant differences between means (P<0.05).
(ZIP)

File S2 **Sequences retrieved from the gilthead sea bream skeletal muscle transcriptome for qPCR primer design.** Sequence identity and e-value were obtained by Blastx against the NCBI non-redundant database and mean coverage was estimated directly from the gilthead sea bream fast skeletal muscle transcriptome assembly.
(TXT)
