# Supplementary figures and images for: Development Temperature Has Persistent Effects on Muscle Growth Responses in Gilthead Sea Bream
Source: PLoS One. 2012 Dec 17;7(12):e51884. doi: 10.1371/journal.pone.0051884 (PMC3524095; doi:10.1371/journal.pone.0051884)

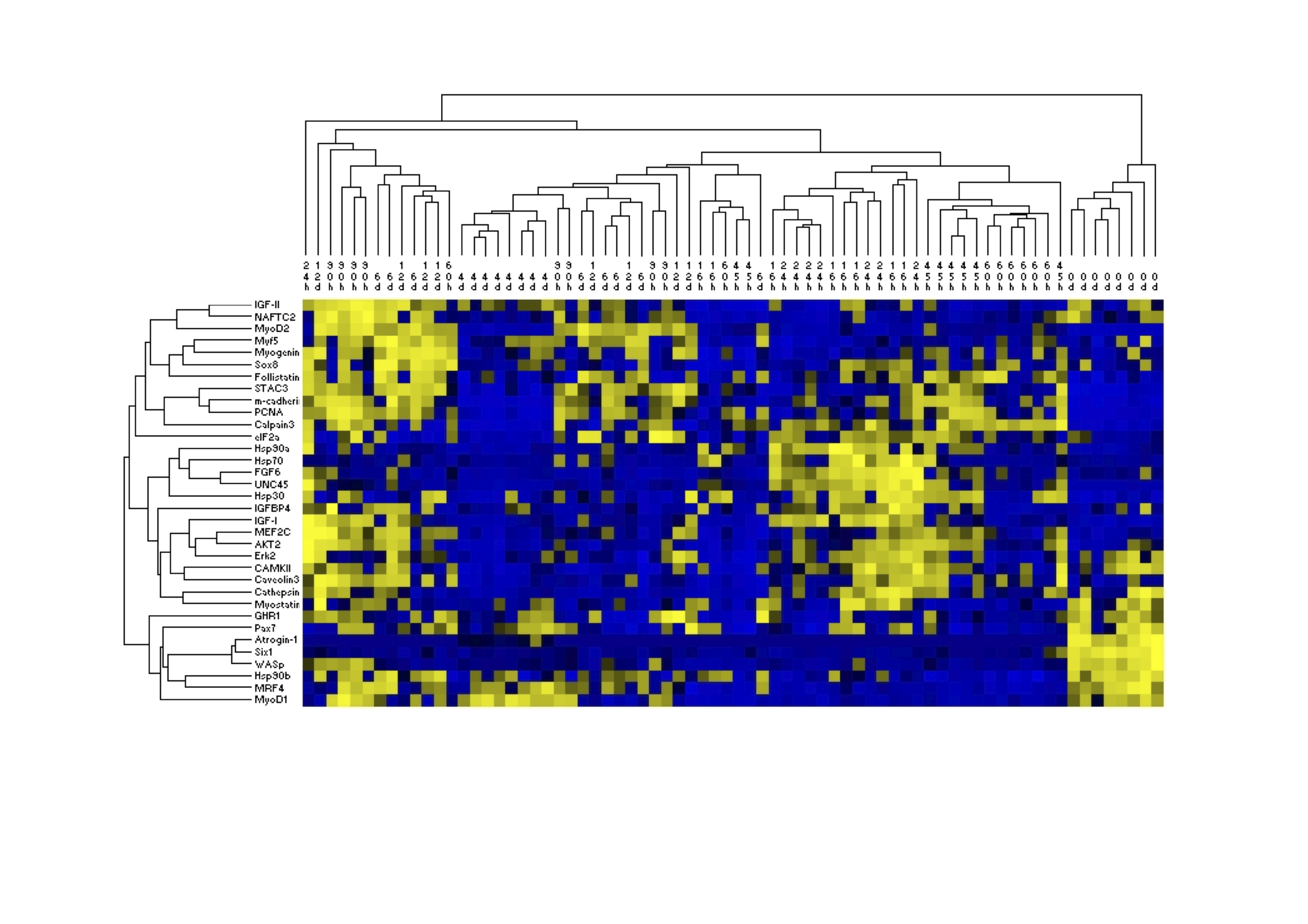

Supplement: Figure S2 — Heat map summary and hierarchical cluster for the 34 genes analysed in fast muscle. Unsupervised hierarchical cluster of 34 genes during fasting and refeeding. Data was clustered by “expression” and “time-point”. Rows are standardized to have a mean of 0 and standard deviation of 1; yellow indicates high and blue indicates low expression values. Insulin-like growth factor 1 (IGF1), Myostatin (MSTN), myoblast determination factor 2 (MyoD2), growth hormone receptor 1 (GHR1), myogenic regulator factor 4 (MRF4), insulin-like growth factor 2 (IGF2), paired box transcription factor 7 (Pax7), myogenic factor 5 (Myf5), sex determination region Y box 8 (Sox8), myogenic regulator factor 1 (MyoD1), heat shock protein 70 (Hsp70), heat shock protein 30 (Hsp30), heat shock protein 90 alpha (Hsp90α), heat shock protein 90 beta (Hsp90β), SH3 and cysteine rich domain 3 (STAC3), proliferating cell nuclear antigen (PCNA), insulin-like growth factor binding protein 4 (IGFBP4), mitogen activated protein kinase (Erk2), v-akt murine thymoma viral oncogene homolog 2 (AKT2), muscle cadherin/cadherin 15 (m-cadherin), myocyte enhancer factor 2c (MEF2C), nuclear factor of activated T-cells calcineurin depenent 2 (NFATC2), F-box protein 32 (MAFbx), eukaryotic initiation translation factor 2a (eIF2a), Wisskott-Aldrich syndrome protein (WASp), sine oculis homeobox 1 (Six1), fibroblast growth factor 6 (FGF6) and calcium/calmodulin-dependent protein kinase 2 (CAMKII). (TIFF) [file pone.0051884.s002.tiff]
