# Supplementary material for: Development Temperature Has Persistent Effects on Muscle Growth Responses in Gilthead Sea Bream
Source: PLoS One. 2012 Dec 17;7(12):e51884. doi: 10.1371/journal.pone.0051884 (PMC3524095; doi:10.1371/journal.pone.0051884)
Supplement: Table S1 — Multivariate statistical analysis of body morphological and gene expression parameters in gilthead sea bream reared at either 17.5–18.5°C (LT) or 21–22°C (HT) until 101d post-hatching and then transferred to a common temperature of 21–22°C. Dependent variables were hepatosomatic index (HSI), mesenteric fat index (MFI), UNC45, heat shock protein 90-alpha (Hsp90α), MAFbx, sine oculis homeobox homolog (Six1), Insulin-like growth factor 1 (IGF1) and myoblast determination protein (MyoD2) expression. Temperature was used as a fixed factor, gut content and time were considered as co-variables with Bonferroni correction for the confidence intervals. (DOCX) [file pone.0051884.s007.docx]

| Factor | Variable | P-value |
| --- | --- | --- |
| *Gut content* | HSI | 0.18 |
|  | MFI | 0.27 |
|  | *MAFbx* | 0.00 |
|  | *Hsp90α* | 0.22 |
|  | *UNC45* | 0.27 |
|  | *IGF1* | 0.74 |
|  | *Six1* | 0.56 |
|  | *MyoD2* | 0.00 |
| *Time* | HSI | 0.00 |
|  | MFI | 0.00 |
|  | *MAFbx* | 0.00 |
|  | *Hsp90α* | 0.00 |
|  | *UNC45* | 0.49 |
|  | *IGF1* | 0.00 |
|  | *Six1* | 0.00 |
|  | *MyoD2* | 0.00 |
| *Temperature* | HSI | 0.13 |
|  | MFI | 0.02 |
|  | *MAFbx* | 0.70 |
|  | *Hsp90α* | 0.00 |
|  | *UNC45* | 0.00 |
|  | *IGF1* | 0.00 |
|  | *Six1* | 0.50 |
|  | *MyoD2* | 0.01 |
