# Supplementary material for: Development Temperature Has Persistent Effects on Muscle Growth Responses in Gilthead Sea Bream
Source: PLoS One. 2012 Dec 17;7(12):e51884. doi: 10.1371/journal.pone.0051884 (PMC3524095; doi:10.1371/journal.pone.0051884)
Supplement: Table S2 — Primer design and qPCR parameters. Forward and reverse primer sequences (5′–3′), amplicon product sizes in base pairs (bp), melting temperature of the amplicon (Tm), PCR efficiency (E), regression analysis of plasmid dilution series (R2) and identification of genes used in qPCR. Genes are as follow: Ribosomal protein L27 (RPL27), mitochondrial ribosomal protein S18 (S18), elongation factor 1-alpha (EF1a), insulin-like growth factor 1 (IGF1), Myostatin (MSTN), myoblast determination factor 2 (MyoD2), growth hormone receptor 1 (GHR1), myogenic regulator factor 4 (MRF4), insulin-like growth factor 2 (IGF2), paired box transcription factor 7 (Pax7), myogenic factor 5 (Myf5), sex determination region Y box 8 (Sox8), myogenic regulator factor 1 (MyoD1), heat shock protein 70 (Hsp70), heat shock protein 30 (Hsp30), heat shock protein 90 alpha (Hsp90α), heat shock protein 90 beta (Hsp90β), SH3 and cysteine rich domain 3 (STAC3), proliferating cell nuclear antigen (PCNA), insulin-like growth factor binding protein 4 (IGFBP4), mitogen activated protein kinase (Erk2), v-akt murine thymoma viral oncogene homolog 2 (AKT2), muscle cadherin/cadherin 15 (m-cadherin), myocyte enhancer factor 2c (MEF2C), nuclear factor of activated T-cells calcineurin depenent 2 (NFATC2), F-box protein 32 (MAFbx), eukaryotic initiation translation factor 2a (eIF2a), Wisskott-Aldrich syndrome protein (WASp), sine oculis homeobox 1 (Six1), fibroblast growth factor 6 (FGF6) and calcium/calmodulin-dependent protein kinase 2 (CAMKII), muscle specific calcium activated neutral protease 3 (Calpain-3), protein unc-45 (UNC45). (DOCX) [file pone.0051884.s008.docx]

| Gene Name | Primers (5’-3’) | E (%) | R^2^ | Size (bp) | Tm (Cº) | Accession number |
| --- | --- | --- | --- | --- | --- | --- |
|  |  |  |  |  |  |  |
| *Actine* | F: TCCTGCGGAATCCATGAGA | 97 | 0.99 | 50 | 76.8 | X89920 |
|  | R: GACGTCGCACTTCATGATGCT |  |  |  |  |  |
| *RPL27* | F:AAGAGGAACACAACTCACTGCCCCAC | 98 | 0.99 | 134 | 86.5 | AY188520 |
|  | R: GCTTGCCTTTGCCCAGAACTTTGTAG |  |  |  |  |  |
| *18S* | F: CGAGCAATAACAGGTCTGTG | 104 | 0.99 | 495 | 85.8 | Castellana et al. 2008 |
|  | R: GGGCATGGACTTAATCAA |  |  |  |  |  |
| *EF1a* | F: CTTCAACGCTCAGGTCATCAT | 97 | 0.99 | 152 | 85.6 | AF184170 |
|  | R: GCACAGCGAAACGACCAAGGGGA |  |  |  |  |  |
| *IGF1* | F: TGTCTAGCGCTCTTTCCTTTCA | 101 | 0.99 | 83 | 79 | AY996779 |
|  | R: AGAGGGTGTGGCTACAGGAGATAC |  |  |  |  |  |
| *MSTN* | F: GTACGACGTGCTGGGAGACG | 99 | 0.99 | 201 | 84.8 | AF258448.1 |
|  | R: CGTACGATTCGATTCGCTTG |  |  |  |  |  |
| *MYOD2* | F: CACTACAGCGGGGATTCAGAC | 100 | 0.99 | 149 | 83.3 | AF478569 |
|  | R: CGTTTGCTTCTCCTGGACTC |  |  |  |  |  |
| *GHR1* | F: ACCTGTCAGCCACCACATGA | 98 | 0.99 | 98 | 84.3 | AF438176 |
|  | R: TCGTGCAGATCTGGGTCGTA |  |  |  |  |  |
| *MRF4* | F: CATCCCACAGCTTTAAAGGCA | 94 | 0.98 | 150 | 84.5 | JN034421 |
|  | R: CATCCCACAGCTTTAAAGGCA |  |  |  |  |  |
| *Myogenin* | F:CAGAGGCTGCCCAAGGTCGAG | 95 | 0.97 | 214 | 87.1 | EF462191 |
|  | R:CAGGTGCTGCCCGAACTGGGCTCG |  |  |  |  |  |
| *IGF2* | F:TGGGATCGTAGAGGAGTGTTGT | 85 | 0.98 | 108 | 83.8 | AY996778 |
|  | R:CTGTAGAGAGGTGGCCGACA |  |  |  |  |  |
| *PAX7* | F:CAGGGTCCAGGTGTGGTTC | 95 | 0.98 | 189 | 83.5 | JN034418 |
|  | R:GGCAACAGGTGGTTAAATGC |  |  |  |  |  |
| *Myf5* | F:GCATGGTTGACAGCAACAGT | 90 | 0.99 | 185 | 79 | JN034420 |
|  | R:CTTATCGCCCAAAGTGTCGT |  |  |  |  |  |
| *Sox8* | F:AACATGCAGGGCAGCCTC | 96 | 0.98 | 116 | 88 | JN034419 |
|  | R:CCCTCGTGCTTCAGATCCT |  |  |  |  |  |
| *MyoD1* | F:GTTTTGTTCCAGGCGGTCT | 97 | 0.98 | 105 | 84.6 | AF478569 |
|  | R:GCTGGTGTCGGTGGAGAT |  |  |  |  |  |
| *Hsp70* | F:TGTGAGAGGGCCAAGAGAAC | 96 | 0.99 | 151 | 84.5 | DQ524995.1+ HS982959.1 |
|  | R:CCCTGGTGATGGAGGTGTAG |  |  |  |  |  |
| *Hsp30* | R:GGTGACTGACGGGAAAGAGA | 94 | 0.98 | 110 | 83.3 | GU060312 |
|  | F:CTGAGGAGGAGGTGCTGTTC |  |  |  |  |  |
| *Follistatin* | R:GCAAAGAAACCTGCGACAAT | 98 | 0.99 | 108 | 83.8 | AY544167 |
|  | F:GATGTTGGAGCAGTCTGGTG |  |  |  |  |  |
| *Hsp90β* | R: TTCACGCATGGAAGAAGTTG | 95 | 0.99 | 99 | 77.9 | Isotig02074 |
|  | F:GGTCCACCACACAACATGAA |  |  |  |  |  |
| *Hsp90α* | F:CGACGACACATCAAGAATGG | 97 | 0.99 | 116 | 77.2 | Contig08719 |
|  | R:GAAATGTCGATTTGAAGAAGCA |  |  |  |  |  |
| *STAC3* | F:CACAGTGATCTCCTCCAGCA | 95 | 0.99 | 152 | 82 | Isotig05310 |
|  | R:CCTTTGTCGGGAACAGAGAG |  |  |  |  |  |
| *PCNA* | F:GAGCAGCTGGGTATTCCAGA | 96 | 0.99 | 149 | 85.4 | Isotig07601 |
|  | R:CTGTGGCGGAGAACTTGACT |  |  |  |  |  |
| *IGFBP4* | F:TCCACAAACCAGAGAAGCAA | 94 | 0.98 | 141 | 85 | F5T95CD02JMZ9K |
|  | R:GGGTATGGGGATTGTGAAGA |  |  |  |  |  |
| *Erk2* | F:AAAGCTCTGGACCTGTTGGA | 101 | 0.98 | 158 | 84.3 | Isotig08986 |
|  | R:TCATCCAGCTCCATGTCAAA |  |  |  |  |  |
| *AKT2* | F:GcTCACCCCACTCTTCAGAC | 97 | 0.99 | 155 | 83 | Isotig05578 |
|  | R: AAATTGGAAATGTGCTTGC |  |  |  |  |  |
| *m-cadherin* | F:GGCCTTCGTGGGTAGATAGG | 98 | 0.99 | 148 | 85.5 | Isotig02137 |
|  | R:TTTAACGTCGACCTGCTGTG |  |  |  |  |  |
| *MEF2c* | F:ACAGCTTGTTGGTGCTGTTG | 95 | 0.98 | 149 | 84.2 | Isotig02218 |
|  | R:AGATAGCGCGAATCATGGAC |  |  |  |  |  |
| *UNC45* | F:CCGCACAACCCTAAAGATG | 98 | 0.99 | 147 | 81.7 | Isotig00833 |
|  | R:TCCTGTGAATGACAGCAAGC |  |  |  |  |  |
| *CathepsinD1* | F:GCGCGTCAAGGTAGTTCTTC | 102 | 0.99 | 148 | 84.3 | Isotig02725 |

|  | R:AAATTCCGTTCCATCAGACG |  |  |  |  |  |
| --- | --- | --- | --- | --- | --- | --- |

| *NFATC2* | F:AGAGCAGCTGTCCATGTCCT | 97 | 0.98 | 145 | | 85.4 | | F5T95CD02I1BGT |
| --- | --- | --- | --- | --- | --- | --- | --- | --- |
|  | R:CGTTCGTCTTGTGTTTCGTG |  |  | |  | |  |  |
| *MAFbx* | F:GGTGCAACTTTCTGGGTTGT | 90 | 0.99 | 105 | | 85 | | Isotig01719 |
|  | R:GGTCACCTGGAGTGGAAGAA |  |  |  | |  | |  |
| *eIF2a* | F:GGAGTGAGTCGCCTGTTGAT | 98 | 0,98 | 154 | | 82.8 | | Isotig06735 |
|  | R:CCTGGGTCTTCGATGAGAAA |  |  |  | |  | |  |
| *Calpain-3* | F:GGCTCTGATGCAGAAAGGTC | 98 | 0.99 | 154 | | 82.5 | | Isotig02937 |
|  | R:TTTAGCCTTGGAGGCTGTGT |  |  |  | |  | |  |
| *WASp* | F:TGTAAGTTCGCCGTCACTTG | 99 | 0.99 | 161 | | 77.5 | | Isotig03137 |
|  | R:ATGTCCGCCACACATGTAAA |  |  |  | |  | |  |
| *Six1* | F:CTGGAGGTTAGAGGTCCCAAC | 97 | 0.99 | 140 | | 88.1 | | Isotig02235 |
|  | R:CTTTTGCTTCAGGGCAACAT |  |  |  | |  | |  |
| *FGF6* | F:TTGCTCATATTCGGGGGTAG | 95 | 0.99 | 149 | | 84.3 | | Isotig12027 |
|  | R:AGCTTGCTCCCGAACAACTA |  |  |  | |  | |  |
| *Caveolin-3* | F:TGTCTGCAGTCTTTGGCATC | 96 | 0.99 | 164 | | 85 | | Isotig05855 |
|  | R:ATCGCAGAAGGTTTGAATGC |  |  |  | |  | |  |
| *CAMKII* | F:GGACAATGTTGGGGTGTTTC | 95 | 0.99 | 160 | | 82.2 | | Isogit05575 |
|  | R:AAGGGGTCCTTTTCAGTGGT |  |  | |  | |  |  |
